# Blockade of JAK2 protects mice against hypoxia‐induced pulmonary arterial hypertension by repressing pulmonary arterial smooth muscle cell proliferation

**DOI:** 10.1111/cpr.12742

**Published:** 2020-01-14

**Authors:** Lei Zhang, Yi Wang, Guorao Wu, Lizong Rao, Yanqiu Wei, Huihui Yue, Ting Yuan, Ping Yang, Fei Xiong, Shu Zhang, Qing Zhou, Zhishui Chen, Jinxiu Li, Bi‐Wen Mo, Huilan Zhang, Weining Xiong, Cong‐Yi Wang

**Affiliations:** ^1^ Key Laboratory of Organ Transplantation NHC Key Laboratory of Organ Transplantation Key Laboratory of Organ Transplantation Ministry of Education The Center for Biomedical Research, Chinese Academy of Medical Sciences Tongji Hospital Tongji Medical College Huazhong University of Science and Technology Wuhan China; ^2^ Department of Respiratory and Critical Care Medicine Tongji Hospital Tongji Medical College Huazhong University of Science and Technology Wuhan China; ^3^ Department of Respiratory and Critical Care Medicine Affiliated Hospital of Guilin Medical University Guilin China; ^4^ Shenzhen Third People's Hospital Shenzhen China; ^5^ Department of Respiratory Medicine Shanghai Ninth People's Hospital Shanghai Jiaotong University School of Medicine Shanghai China

**Keywords:** cyclin A2, Janus kinase 2, pulmonary arterial hypertension, pulmonary artery smooth muscle cell, signal transducer and activator of transcription 3

## Abstract

**Objectives:**

Hypoxia is an important risk factor for pulmonary arterial remodelling in pulmonary arterial hypertension (PAH), and the Janus kinase 2 (JAK2) is believed to be involved in this process. In the present report, we aimed to investigate the role of JAK2 in vascular smooth muscle cells during the course of PAH.

**Methods:**

Smooth muscle cell (SMC)‐specific *Jak2* deficient mice and their littermate controls were subjected to normobaric normoxic or hypoxic (10% O_2_) challenges for 28 days to monitor the development of PAH, respectively. To further elucidate the potential mechanisms whereby JAK2 influences pulmonary vascular remodelling, a selective JAK2 inhibitor was applied to pre‐treat human pulmonary arterial smooth muscle cells (HPASMCs) for 1 hour followed by 24‐hour hypoxic exposure.

**Results:**

Mice with hypoxia‐induced PAH were characterized by the altered JAK2/STAT3 activity in pulmonary artery smooth muscle cells. Therefore, induction of *Jak2* deficiency in SMCs protected mice from hypoxia‐induced increase of right ventricular systolic pressure (RVSP), right ventricular hypertrophy and pulmonary vascular remodelling. Particularly, loss of *Jak2* significantly attenuated chronic hypoxia‐induced PASMC proliferation in the lungs. Similarly, blockade of JAK2 by its inhibitor, TG‐101348, suppressed hypoxia‐induced human PASMC proliferation. Upon hypoxia‐induced activation, JAK2 phosphorylated signal transducer and activator of transcription 3 (STAT3), which then bound to the *CCNA2* promoter to transcribe cyclin A2 expression, thereby promoting PASMC proliferation.

**Conclusions:**

Our studies support that JAK2 could be a culprit contributing to the pulmonary vascular remodelling, and therefore, it could be a viable target for prevention and treatment of PAH in clinical settings.

## 1 INTRODUCTION

Pulmonary arterial hypertension (PAH) is a life‐threatening disease manifested by the progressive pulmonary vascular remodelling, which results in persistently increased pulmonary arterial pressure, eventually culminating in right heart failure.^1^, ^2^ Sustained pulmonary vasoconstriction and vascular remodelling characterized by the concentric wall thickening and lumen obliteration of small‐ and medium‐sized pulmonary arteries (PAs), are the major causes of elevated pulmonary vascular resistance and pulmonary arterial pressure in patients with PAH.^3^ There is compelling evidence that pulmonary arterial smooth muscle cell (PASMC) hyperplasia is a cardinal feature of pulmonary vascular remodelling that underlies the development and progression of PAH.^4^, ^5^ A constitutive proliferative phenotype in PASMCs is considered to be a critical feature for patients with PAH or animals with induced PAH.^6^, ^7^ As a result, although current therapies against PAH can improve clinical symptoms, but disease progression is inevitable in most patients and mortality remains unacceptably high.^8^, ^9^ Therefore, new therapies aimed at ameliorating the irreversible pulmonary arterial remodelling are wanting in clinical settings.

As non‐receptor tyrosine kinases, the mammalian Janus kinase (JAK) family comprises four evolutionarily conserved members, JAK1, JAK2, JAK3 and tyrosine kinase 2 (TYK2). Upon engagement of cytokines with cell surface receptors, JAKs undergo autophosphorylation at tyrosine residues, which generates docking sites to phosphorylate signal transducers and activators of transcription (STAT). Once STATs are phosphorylated at highly conserved tyrosine residues (termed p‐STAT) by JAKs or other tyrosine kinases, they form dimers and then translocate into the nucleus, where they bind to the specific promoters to transcript the genes involved in cell proliferation, differentiation and apoptosis.^10^, ^11^ As an important member of the JAK family, Janus kinase 2 (JAK2) is involved in the regulation of various processes relevant to cell survival, proliferation, activation and differentiation. Particularly, JAK2 gain‐of‐function somatic mutations have been characterized in most patients with myeloproliferative neoplasms (MPNs) and acute lymphoblastic leukemia (ALL).^12^, ^13^, ^14^ As a result, JAK inhibitors have been developed to treat various malignancies and have been shown to be efficacious against solid tumours, psoriasis and rheumatoid arthritis in both preclinical and clinical settings.^15^, ^16^, ^17^

The functional importance of JAK2 in systemic arterial vascular smooth muscle cells (VSMCs) has also been noted,^18^ but its role in VSMCs during the course of pulmonary blood vessel remodelling is yet to be clarified. To address this question, we generated a SMC‐specific *Jak2* knockout model and demonstrated that *Jak2* deficiency in SMCs protected mice from hypoxia‐induced PAH and substantially reduced right ventricular systolic pressure (RVSP), the right ventricle/left ventricle plus septum [RV/ (LV+S)] weight ratio and the median width of pulmonary arterioles. Mechanistic studies revealed that blockade of JAK2 activity inhibited hypoxia‐induced HPASMC proliferation by repressing the binding activity of STAT3 to the *CCNA2* promoter, thereby attenuating pulmonary blood vessel remodelling. Our data support that strategies aimed at inhibiting JAK2 activity could be a viable treatment for PAH in clinical settings.

## 2 MATERIAL AND METHODS

### 2.1 Mice

*Jak2^flox/flox^ (H‐2^b^)* mice were generated as described previously.^19^ *SM22‐Cre^ERT2^* mice were crossed with *Jak2^flox/flox^* mice to generate *Jak2^flox/flox^SM22‐Cre^ERT2+^* mice. *Jak2* deficiency (*Jak2* conditional knockout (*Jak2‐CKO*)) in *Jak2^flox/flox^SM22‐Cre^ERT2+^* mice was induced by intraperitoneal (i.p.) injection of tamoxifen (75 mg/kg; Sigma) for five consecutive days. Littermates (*Jak2^flox/flox^SM22‐Cre^ERT2−^* mice) administered with equal dose of tamoxifen were used as controls (*Jak2‐C*). Wild‐type (WT) C57BL/6 mice were purchased from the Jackson's Laboratory (Bar Harbor). Eight‐week‐old male mice were used for the experimental purposes. All mice were housed at the Tongji Medical College Animal Center with a 12/12‐hour light/dark cycle (Wuhan, China) in a specific pathogen‐free (SPF) facility.

### 2.2 Antibodies

Antibodies against JAK2 (Cat. No. #3230s), STAT3 (Cat. No. #79D7), phospho‐STAT3 (Tyr705) (Cat. No. #9145s) and phospho‐STAT3 (Tyr705) (Cat. No. #4113s) were purchased from the Cell Signaling Technology. Phospho‐JAK2 (Tyr1007) (Cat. No. ab195055) antibody was purchased from the Abcam. Cyclin A2 (Cat. No. 18202‐1‐AP), and beta‐actin (Cat. No. HRP‐60008) antibodies were obtained from the Proteintech Group. Alpha‐smooth muscle actin (α‐SMA) (Cat. No. #Sc‐53015) and Ki67 (Cat. No. #Sc‐7846) antibodies were from the Santa Cruz Biotechnology.

### 2.3 Exposure to chronic hypoxia

Eight‐week‐old male mice were exposed to normobaric normoxia (inspired O_2_ fraction [FiO_2_] of 0.21) or normobaric hypoxia (FiO_2_ of 0.10) for 28 days. Briefly, the animals were divided randomly into four groups: (a) *Jak2‐C* normoxic group (21% O_2_, n = 8); (b) *Jak2‐CKO* normoxic group (21% O_2_, n = 8); (c) *Jak2‐C* hypoxic group (10% O_2_, n = 10); and (d) *Jak2‐CKO* hypoxic group (10% O_2_, n = 10).

### 2.4 Cell culture

Human pulmonary arterial smooth muscle cells were purchased from the ScienCell and cultured with smooth muscle cell growth medium 2 (SMCGM 2, PromoCell) supplemented with 10% foetal bovine serum (FBS), 100 mg/mL penicillin and 100 IU/mL streptomycin at 37°C in a humidified atmosphere of 5% CO_2_ in air. Cells at passages 4‐9 were used for the experiments. For hypoxia (2% O_2_) experiments, cells were firstly starved for 12 hours and then placed in a HERAcell vios 160i CO_2_ incubator (Thermo Fisher), which was infused with a gas mixture containing 5% CO_2_ and 93% N_2_ for 24 hours. Normal incubators with 21% O_2_ were used for normoxic culture. JAK2 phosphorylation was blocked in human PASMCs by adding 1 µmol/L TG‐101348 (fedratinib MedChemExpress) 1 hour prior to hypoxic exposure. JAK2 phosphorylation was then assessed at the indicated time points.

### 2.5 Hemodynamic measurements

After normoxic or hypoxic exposure, mice were anaesthetized with sodium pentobarbital (60 mg/kg), and hemodynamic measurements were performed. Measurement of RVSP and systemic arterial pressure was performed as described previously.^20^ After exsanguination, the left lungs were fixed for histology in 4% neutral buffered formalin, and the right lungs were snap‐frozen. The right ventricle (RV) was separated from the left ventricle plus septum (LV+S), and the RV/(LV+S) ratio was calculated as an index of RV hypertrophy. Lung vascular remodelling was assessed by measuring the degree of vessel muscularization as reported.^20^

### 2.6 Western blot analysis

Total protein was isolated from cultured cells using RIPA buffer (Beyotime), and the concentration was quantified using a BCA Protein Assay Kit (Boster). The proteins were subjected to Western blotting with the indicated primary antibodies using the established techniques.^21^, ^22^

### 2.7 Quantitative RT‐PCR analysis

Quantitative RT‐PCR analysis was conducted using the SYBR Premix Ex Taq (TaKaRa) as previously described.^23^ Briefly, total RNA was extracted from HPASMCs using a RNAiso plus kit (TaKaRa) according to the supplier's instructions. Real‐time RT‐PCR was conducted to assess cyclin A2, cyclin D1, cyclin E1, CDK2 and CDK4 expression using an ABI prisDK1m 7500 Sequence Detection System (Applied Biosystems). β‐actin was used for normalization, and the relative expression levels for each target gene were calculated using the 2^−ΔΔCt^ approach as previously reported.^24^ The primers used to amplify each target gene were as follows: human *CCNA2*: forward: 5′‐CGC TGG CGG TAC TGA AGT C‐3′, reverse: 5′‐GAG GAA CGG TGA CAT GCT CAT‐3′; human *CCND1*: forward: 5′‐GCT GCG AAG TGG AAA CCA TC‐3′, reverse: 5′‐CCT CCT TCT GCA CAC ATT TGA A‐3′; human *CCNE1*: forward: 5′‐AAG GAG CGG GAC ACC ATG A‐3′, reverse: 5′‐ACG GTC ACG TTT GCC TTC C‐3′; human *CDK2*: forward: 5′‐CCA GGA GTT ACT TCT ATG CCT GA‐3′, reverse: 5′‐TTC ATC CAG GGG AGG TAC AAC‐3′; human *CDK4*: forward: 5′‐ATG GCT ACC TCT CGA TAT GAG C‐3′, reverse: 5′‐CAT TGG GGA CTC TCA CAC TCT‐3′; human *beta‐actin*: forward: 5′‐CAT GTA CGT TGC TAT CCA GGC‐3′, reverse: 5′‐ CTC CTT AAT GTC ACG CAC GAT‐3′. Human *beta‐actin* was used as an internal control.

### 2.8 Histological and immunohistochemical analysis

The lungs were fixed by intratracheal infusion of 4% aqueous buffered formalin. Midsagittal slices from the right lungs were processed for paraffin embedding and sliced into 5‐µm sections. The sections were next subjected to haematoxylin and eosin (HE) and elastic van gieson (EVG) staining using the established techniques.^25^, ^26^ For immunofluorescence analysis, fresh frozen sections (7 µm) of lung tissues and cells grown on coverslips in the chamber slides were prepared as previously described.^27^, ^28^ The slides were co‐incubated with primary antibodies against p‐STAT3/α‐SMA, Ki67/α‐SMA, cyclin A2/α‐SMA and p‐JAK2/p‐STAT3, followed by probing with Alexa 594‐labelled or Alexa 488‐labelled secondary antibodies (Invitrogen). The tissue slides were assessed by two pathologists using a brightfield or fluorescence microscope (Olympus) in a blinded fashion. The cell slides were analysed under a laser‐scanning confocal microscope (LSM 510 Meta Zeiss). Immunohistochemical staining was performed on paraffin‐embedded slides (5 µm) using a Quick Kit (Beyotime). Paraffin‐embedded serial lung sections were stained with anti‐JAK2 or anti‐α‐SMA antibodies (Ab) (1:200 dilution). Negative controls were prepared by omitting the primary antibody.

### 2.9 Proliferation assays

The cells (approximately 3000 cells/well) were seeded into 96‐well plates and treated with either control vehicle or different concentrations of TG before exposure to hypoxia. After hypoxic exposure for 24 hours, CCK‐8 solution was added following the manufacturer's instructions (Dojindo Laboratories) for analysis of cell proliferation. For carboxyfluorescein diacetate succinimidyl ester (CFSE)‐based proliferation analysis, the cells were harvested and diluted to 1 × 10^6^‐5 × 10^6^ cells/mL in 1 mL of CFSE staining solution (1×) and stained with an equal volume of CFSE stock solution (2×) for 10 minutes at 37°C. The staining was stopped by adding 10 mL of complete SMCGM 2 medium. After washes with complete medium twice, the cells were plated in 6‐well plates at a density of 1 × 10^5^ cells/well, and 1 × 10^5^ cells were left untreated as a positive control. After culture of additional 24 hours, the cells were harvested, and cell proliferation was determined by analysis of the excitation at 488 nm using a flow cytometer (BD Biosciences) with the FACS Express V3 software (De Novo Software). For 5‐ethynyl‐uridine (EdU) staining‐based analysis, HPASMC proliferation was evaluated by determining the ratio of proliferating cells labelled with EdU to the total cell number labelled by Hoechst 33342 staining.^29^

### 2.10 Cell cycle assay

A Cell Cycle and Apoptosis Analysis Kit (Beyotime) was employed for analysis of cell cycles. The synchronized HPASMCs were washed three times with cold PBS and then fixed by 70% ethanol in PBS at −20°C for 12 hours. After washes with cold PBS, the cells were stained with 0.5 mL of propidium iodide (PI) staining buffer (200 mg/mL RNase A and 50 µg/mL PI) at 37°C for 30 minutes in the dark and then subjected to cell cycle analysis using a BD LSR flow cytometer (BD Biosciences).

### 2.11 Chromatin immunoprecipitation (ChIP) assay and CCNA2 promoter reporter assay

ChIP assays were conducted with a ChIP assay kit (Beyotime) as previously described.^25^ The primers used in the ChIP assay were as follows: 5′‐CCG CCC CAG CCA GTT T‐3′ and 5′CCC GCT CGC TCA CCC A‐3′. A dual luciferase reporter system (Promega) was used for the *CCNA2* promoter luciferase reporter assays in which the STAT3 binding site within the *CCNA2* promoter was disrupted using the established techniques.^30^

### 2.12 Statistical analysis

All experiments were conducted with at least three independent replications, and the data are presented as the mean ± SEM. Statistical analysis was performed using the Graph Pad Prism (version 5.0) software (GraphPad Software Inc). Two experimental groups were compared using a Student's *t* test for paired data or a Student's *t* test with Welch's correction for unpaired data. Where more than two groups were compared, a one‐way ANOVA with Bonferroni's correction was used. In all cases, *P* < .05 was considered statistically significant.

## 3 RESULTS

### 3.1 Hypoxic insults activate the JAK2/STAT3 signalling in PASMCs

**Figure 1:**
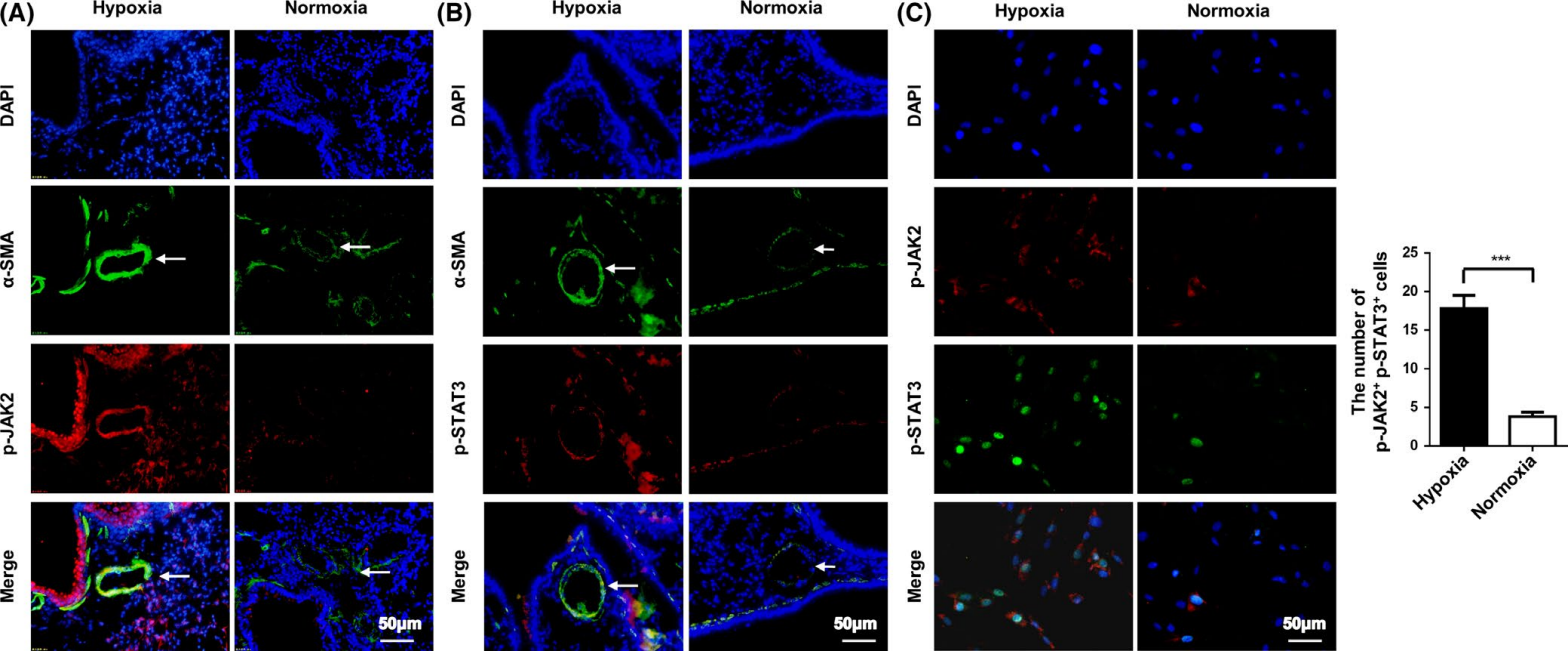
Hypoxia‐induced JAK2/STAT3 activation in a PAH mouse model and HPASMCs. Representative results for coimmunostaining of p‐JAK2 and α‐SMA (A), p‐STAT3 and α‐SMA (B) in lung sections from WT mice exposed to normoxia or hypoxia (n = 3 per group). Confocal immunofluorescence images for coimmunostaining of p‐JAK2 and p‐STAT3 in HPASMCs following normoxic or hypoxic exposure (C). All images were taken at an original magnification of ×400. The data are represented as the mean ± SEM. ***P < .001

We first sought to examine the expression of JAK2 and STAT3 in the lungs of WT C57BL/6 mice with PAH induced by hypoxic challenge (10% O_2_, 28 days). Notably, p‐JAK2 and p‐STAT3 were ubiquitously expressed in the lung sections of mice with PAH. However, this expression was increased mainly in the media layer of the pulmonary arterial region of the PAH lung tissues, as evidenced by the co‐staining of p‐JAK2 or p‐STAT3 with α‐SMA, a marker for PASMCs (Figure 1A,B).

To confirm the above data, we exposed HPASMCs to normoxic or hypoxic conditions. In line with the above observations in animals, a 24 hours of hypoxic challenge (2% O_2_) stimulated a marked increase of JAK2 phosphorylation at Tyr1007 (p‐JAK2) along with an augmented phosphorylated STAT3 at Tyr705 (p‐STAT3) in the nuclei of HPASMCs (Figure 1C; 17.80 ± 1.72 vs 3.80 ± 0.58, n = 5 per group, *P* < .0001). Collectively, these data support that hypoxic insults activate JAK2/STAT3 signalling in PASMCs, which may contribute to the pathogenesis of pulmonary arterial remodelling during the course of PAH development.

### 3.2 Generation of mice with SMC‐specific Jak2 deficiency

**Figure 2:**
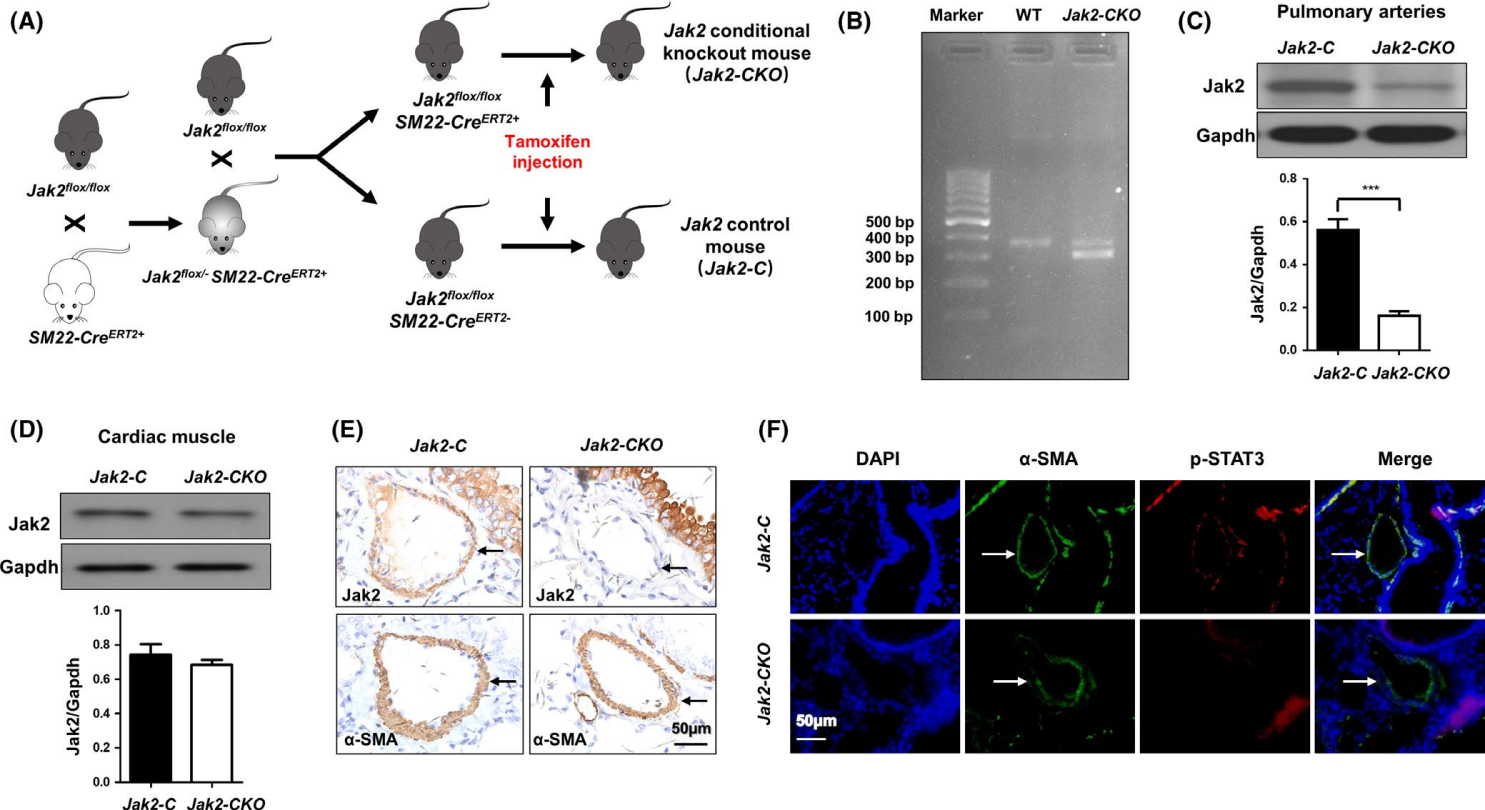
Generation of SMC‐specific *Jak2*‐knockout mice. Schematic diagram of transgenic mice used to generate *Jak2‐CKO* and *Jak2‐C* mice (A). PCR analysis of tail genomic DNA to determine the presence of the floxed null allele (B). Western blot analysis to confirm *Jak2* depletion in pulmonary arteries (C; n = 3 per group) and cardiac muscle (D; n = 3 per group). Immunohistochemistry analysis to confirm *Jak2* depletion in PASMCs (E; n = 3 per group). Coimmunostaining results of p‐STAT3 and α‐SMA in lung sections from *Jak2‐C* and *Jak2‐CKO* mice (F). All images were taken at an original magnification of ×400. The data are represented as the mean ± SEM. ***P < .001

To demonstrate the above assumption, the *Jak2^flox/flox^* mice were bred with *SM22‐Cre^ERT2^* mice to generate *Jak2^flox/flox^SM22‐Cre^ERT2+^* mice (Figure 2A). *Jak2* deficiency (*Jak2‐CKO*) in smooth muscle cells was induced in 8‐week‐old *Jak2^flox/flox^SM22‐Cre^ERT2+^* mice by intraperitoneal injection of tamoxifen for 5 consecutive days, and *Jak2* depletion was confirmed by genotyping tail blood DNA for the presence of the floxed null allele (Figure 2B). Western blot analysis of pulmonary artery lysates also indicated a significant decrease of Jak2 expression (Figure 2C; 0.56 ± 0.05 vs 0.16 ± 0.02, n = 3 per group, *P* < .0001), while its levels in the heart were unaltered (Figure 2D, 0.74 ± 0.06 vs 0.68 ± 0.03, n = 3 per group, *P* = .4040). Immunohistochemical staining of serial lung sections further revealed the absence of Jak2 in PASMCs in *Jak2‐CKO* mice (Figure 2E). Consistently, coimmunostaining demonstrated that the expression of Jak2 downstream target, p‐STAT3, in PASMCs, was substantially lower in *Jak2‐CKO* mice than that in *Jak2‐C* mice (Figure 2F), indicating that tamoxifen efficiently induced *Jak2* deficiency.

### 3.3 Jak2 deficiency alleviates pulmonary pressure and right ventricular function in a hypoxia‐induced mouse model of PAH

**Figure 3:**
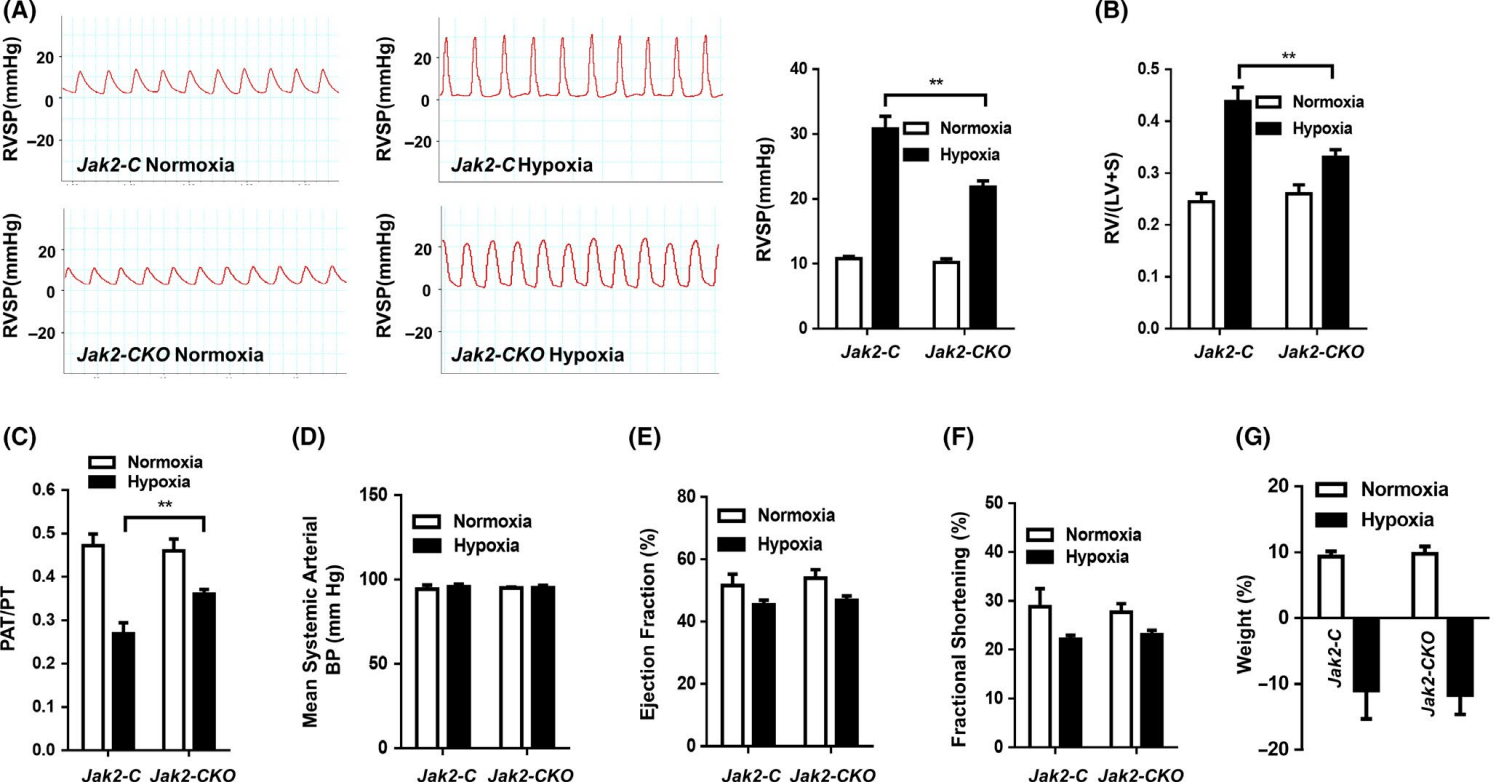
SMC‐specific *Jak2* deficiency improved the reaction of pulmonary blood vessels to hypoxic condition. RVSP (A), RV/(LV + S) ratio (B), PAT/PT ratio (C), mean systemic arterial BP (D), ejection fraction (E), fractional shortening (F) and body weight change ratio (G) in *Jak2‐C* and *Jak2‐CKO* mice after exposure to normoxic (n = 8 per group) or hypoxic (n = 10 per group) conditions for 28 days. The data are represented as the mean ± SEM. **P < .01. RVSP, right ventricular systolic pressure; RV/ (LV+S), the right ventricle/left ventricle plus septum; PAT/PT, pulmonary acceleration time/pulmonary ejection time

Next, *Jak2‐CKO* mice and *Jak2‐C* mice following last tamoxifen injection were subjected to induction of PAH under normobaric normoxic or hypoxic conditions (10% O_2_) for 28 days, respectively. The right ventricular (RV) systolic pressure (RVSP) and RV/(L+S) ratio (the ratio of the right ventricular mass to the sum of the left ventricular and septal masses), a marker of hypertrophy in the right ventricle due to elevated right ventricular pressure and afterload, were employed to assess the development of pulmonary hypertension. After 28 days of hypoxic exposure, the mean RVSP of the hypoxia group was significantly increased compared with that of the normoxia group in both *Jak2‐C* and *Jak2‐CKO* mice. However, the mean RVSP was significantly lower in *Jak2‐CKO* mice than that in *Jak2‐C* mice (Figure 3A; 30.80 ± 1.91 vs 21.80 ± 0.97, n = 10 per group, *P* = .003). In addition, the severity of RV hypertrophy was much lower in *Jak2‐CKO* mice than that in *Jak2‐C* mice after normobaric hypoxic treatment, as determined by the RV/(LV+S) ratio (Figure 3B; 0.44 ± 0.03 vs 0.33 ± 0.01, n = 10 per group, *P* = .002). Similarly, *Jak2‐CKO* mice manifested improvement of impaired right heart function after exposure to chronic hypoxia according to the pulmonary acceleration time/pulmonary ejection time (PAT/PT) ratio (Figure 3C; 0.27 ± 0.03 vs 0.36 ± 0.01, n = 10 per group, *P* = .006).

As expected, echocardiographic analysis revealed that the left heart function did not differ between *Jak2‐CKO* and *Jak2‐C* mice after exposure to normobaric hypoxic condition based on the levels of mean systemic arterial blood pressure (Figure 3D; 95.75 ± 1.39 vs 95.16 ± 1.31, n = 10 per group, *P* = .772), ejection fraction (Figure 3E; 45.41 ± 1.49 vs 46.80 ± 1.43, n = 10 per group, *P* = .523) and fractional shortening (Figure 3F; 22.14 ± 0.78 vs 23.07 ± 0.91, n = 10 per group, *P* = .468). Furthermore, we observed that exposure to chronic hypoxia induced a similar decrease in the total body weight in both the *Jak2‐C* and *Jak2‐CKO* groups (Figure 3G; −10.98 ± 4.343 vs −11.66 ± 2.950, n = 10 per group, *P* = .897). Collectively, our results indicate that *Jak2* deficiency in smooth muscle cells improves the function of pulmonary blood vessels following hypoxic insult.

### 3.4 Jak2 deficiency attenuates pulmonary blood vessel remodelling and PASMC hyperplasia following chronic hypoxia

**Figure 4:**
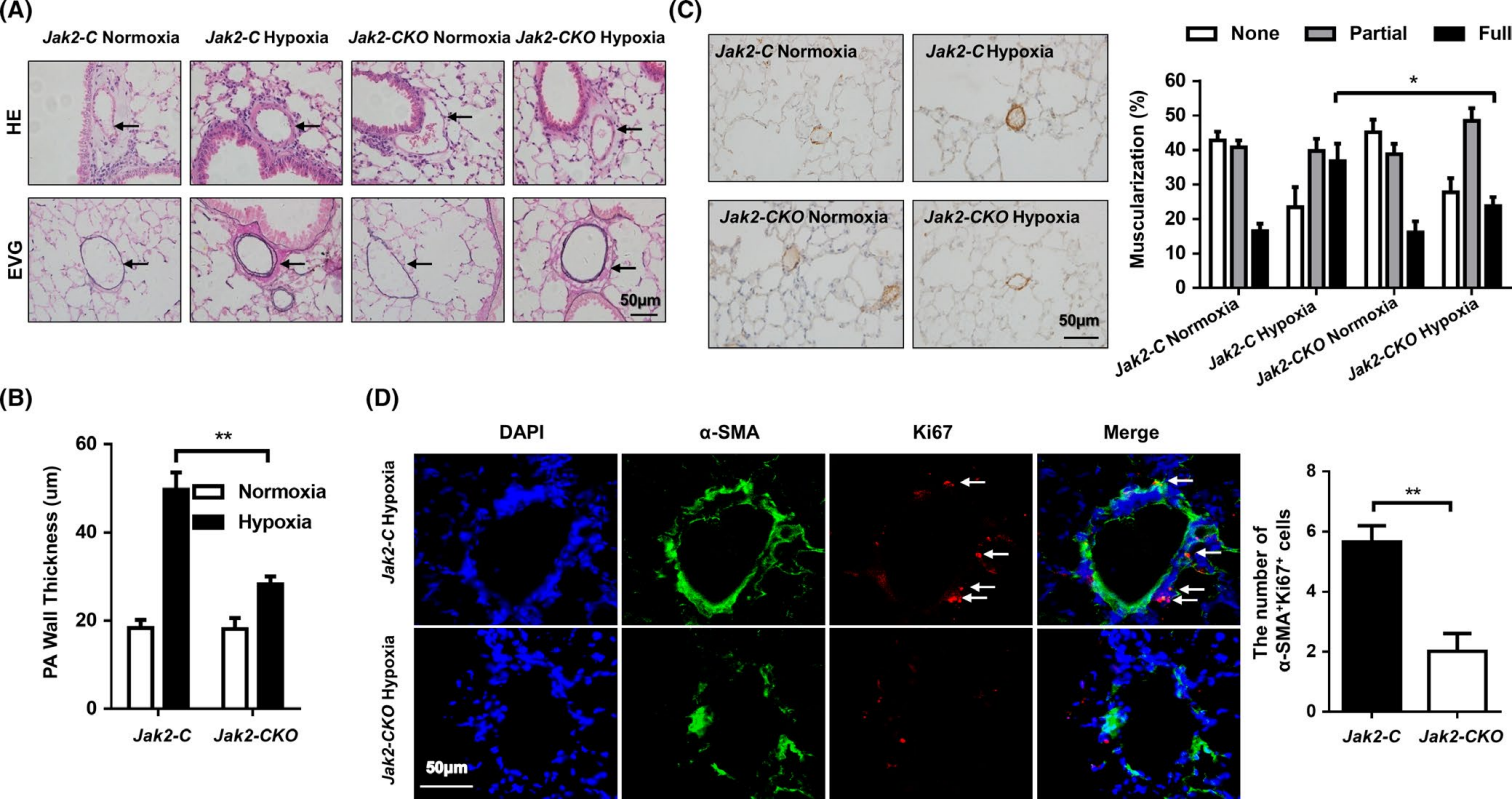
Loss of *Jak2* in smooth muscle cells protected against pulmonary vascular remodelling after hypoxia. Representative HE‐stained (top) and EVG‐stained (bottom) sections (A), quantification of pulmonary arteriole wall thickness (B) and α‐SMA immunostaining (C) in the lungs of *Jak2‐C* and *Jak2‐CKO* mice after normoxic (n = 8 per group) or hypoxic (n = 10 per group) exposure for 28 days. Ten vessels were analysed per mouse. Coimmunostaining results of α‐SMA and Ki67 in lung sections from *Jak2‐C* and *Jak2‐CKO* mice after hypoxic (D; n = 10 per group) exposure for 28 days. All images were taken at an original magnification of ×400. The data are represented as the mean ± SEM. *P < .05; **P < .01. HE, haematoxylin and eosin; EVG, elastic van gieson; PA, pulmonary artery

Histological studies were next conducted to demonstrate the effect of Jak2 on pulmonary blood vessel remodelling during the course of PAH development. Remarkably, staining with haematoxylin and eosin (HE) demonstrated that loss of *Jak2* significantly attenuated hypoxia‐induced blood vessel remodelling (Figure 4A, upper panel), and elastic van gieson (EVG) staining of lung sections further revealed attenuated medial thickening in the small pulmonary arteries in the *Jak2‐CKO* mice (Figure 4A, lower panel). As a result, *Jak2* deficiency significantly reduced pulmonary arteriole wall thickness following chronic hypoxic insult (Figure 4B; 56.81 ± 10.10 vs 30.20 ± 2.62, n = 10 per group, *P* = .002).

To determine whether pulmonary vascular remodelling following chronic hypoxic insult involves PASMC hyperplasia, we first analysed the expression of alpha‐smooth muscle actin (α‐SMA, a marker for smooth muscle cells) in the pulmonary arteries. Histochemical staining of α‐SMA immunostaining revealed that the blood vessels in WT mice were profoundly remodelled in response to chronic hypoxic exposure as manifested by the full neomuscularization of the distal pulmonary arterioles, neointimal enlargement and α‐SMA expressions. In sharp contrast, the PAs originated from *Jak2‐CKO* mice were remodelled to a significantly less degree and harboured much lower α‐SMA expressions (Figure 4C; full muscularization: 36.81 ± 5.05 vs 23.77 ± 2.65, n = 10 per group, *P* = .043). However, the percentage of muscularization of distal PAs was not affected in normoxic *Jak2‐C* and *Jak2‐CKO* mice. Next, we checked PASMC proliferation by co‐immunostaining of α‐SMA and Ki67 in lung sections. Indeed, loss of *Jak2* significantly repressed hypoxia‐induced PASMC proliferation as evidenced by the much less number of α‐SMA and Ki67 double‐positive cells (Figure 4D; 5.65 ± 0.54 vs 2.02 ± 0.59, n = 10 per group, *P* = .002). Thus, loss of *Jak2* in smooth muscle cells may confer protective effects with respect to pulmonary vascular remodelling and PASMC hyperplasia after hypoxic exposure.

### 3.5 JAK2 inhibition suppresses hypoxia‐induced PASMC proliferation

**Figure 5:**
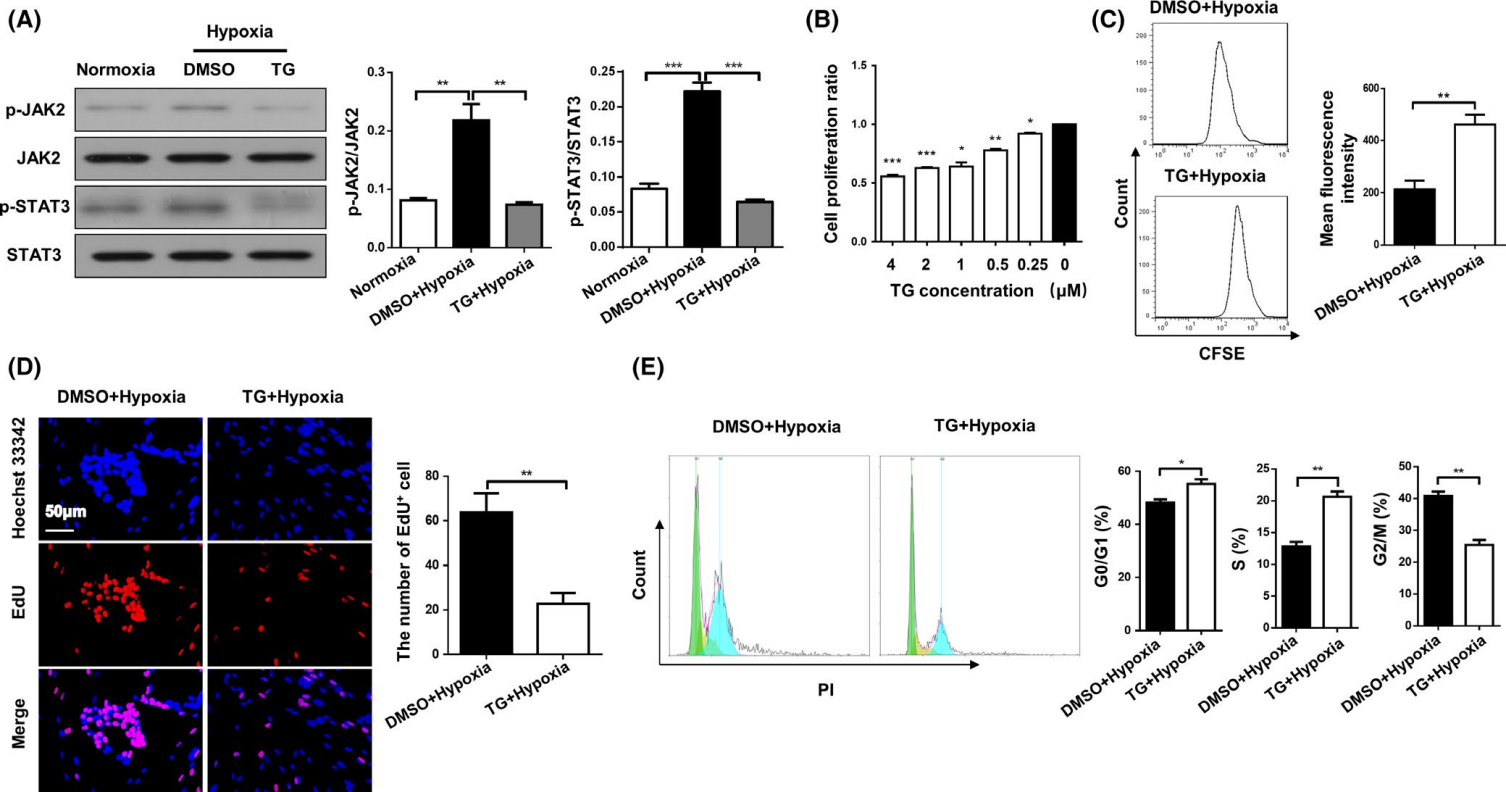
Hypoxia‐induced HPASMC proliferation was suppressed by a JAK2 inhibitor. Western blot analysis of p‐JAK2, JAK2, p‐STAT3 and STAT3 in HPASMCs (A). CCK‐8 analysis of HPASMCs pre‐treated with different concentrations of TG for 1 h following 24 h hypoxic exposure (B). CFSE dilution analysis (C), EdU staining (D) and cell cycle analysis (E) of HPASMCs pre‐treated with DMSO or TG for 1 h following 24 h hypoxic exposure. All images were taken at an original magnification of ×400. The data are represented as the mean ± SEM. *P < .05; **P < .01; ***P < .001. TG, TG‐101348; CFSE, carboxyfluorescein diacetate succinimidyl ester; EdU, 5‐ethynyl‐uridine; DMSO, dimethyl sulphoxide

In general, there is a PASMC phenotype switch from a differentiated state to a proliferative state during the course of PAH development.^31^ To further confirm the above data observed in animals, we employed TG‐101348 (also named Fedratinib, thereafter referred to as TG), a selective JAK2 inhibitor,^32^ to pre‐treat HPASMCs for 1 hour, followed by hypoxic insult for 24 hour. Western blotting revealed that TG did not affect the expression of JAK2, but significantly repressed hypoxia‐induced JAK2 activation (p‐JAK2 levels, 0.22 ± 0.03 vs 0.07 ± 0.00, n = 4 per group, *P* = .002) along with attenuated STAT3 activity (p‐STAT3 levels, 0.22 ± 0.01 vs 0.06 ± 0.00, n = 4 per group, *P* < .0001) (Figure 5A). Next, three complementary approaches were employed to assess the impact of JAK2 on hypoxia‐induced HPASMC proliferation. CCK‐8 assays indicated that TG inhibited PASMC proliferation in a dose‐dependent manner (Figure 5B, 0.25 µmol/L: 0.92 ± 0.01 vs 1.000 ± 1.19 n = 3 per group, *P* = .010). Consistently, carboxyfluorescein diacetate succinimidyl ester (CFSE, 213.30 ± 33.35 vs 461.80 ± 37.85, n = 3 per group, *P* = .003)‐ and 5‐ethynyl‐uridine (EdU, 63.75 ± 8.59 vs 22.75 ± 4.87, n = 3 per group, *P* = .006)‐based assays demonstrated that TG could potently suppressed HPASMC proliferation following hypoxic challenge (Figure 5C,D). Next, we conducted cell cycle analysis and found that TG treatment remarkably reduced the number of 4N DNA (G2/M phase, 40.83 ± 1.34 vs 25.45 ± 1.58, n = 3 per group, *P* = .002) cells following hypoxic induction, while the number of cells containing 2N‐4N DNA (S phase, 12.82 ± 0.74 vs 20.61 ± 0.83, n = 3 per group, *P* = .002) and 2N DNA (G0/G1 phase, 48.29 ± 1.18 vs 55.31 ± 1.69, n = 3 per group, *P* = .027) increased significantly after TG treatment (Figure 5E), suggesting that TG treatment arrested HPASMCs in the G0/G1/S phase following hypoxic induction. Collectively, our data suggest that loss of *Jak2* in smooth muscle cells confers protection against hypoxia‐induced pulmonary hypertension by attenuating pulmonary vascular remodelling and PASMC hyperplasia.

### 3.6 JAK2/STAT3 regulates HPASMC proliferation by enhancing cyclin A2 expression

**Figure 6:**
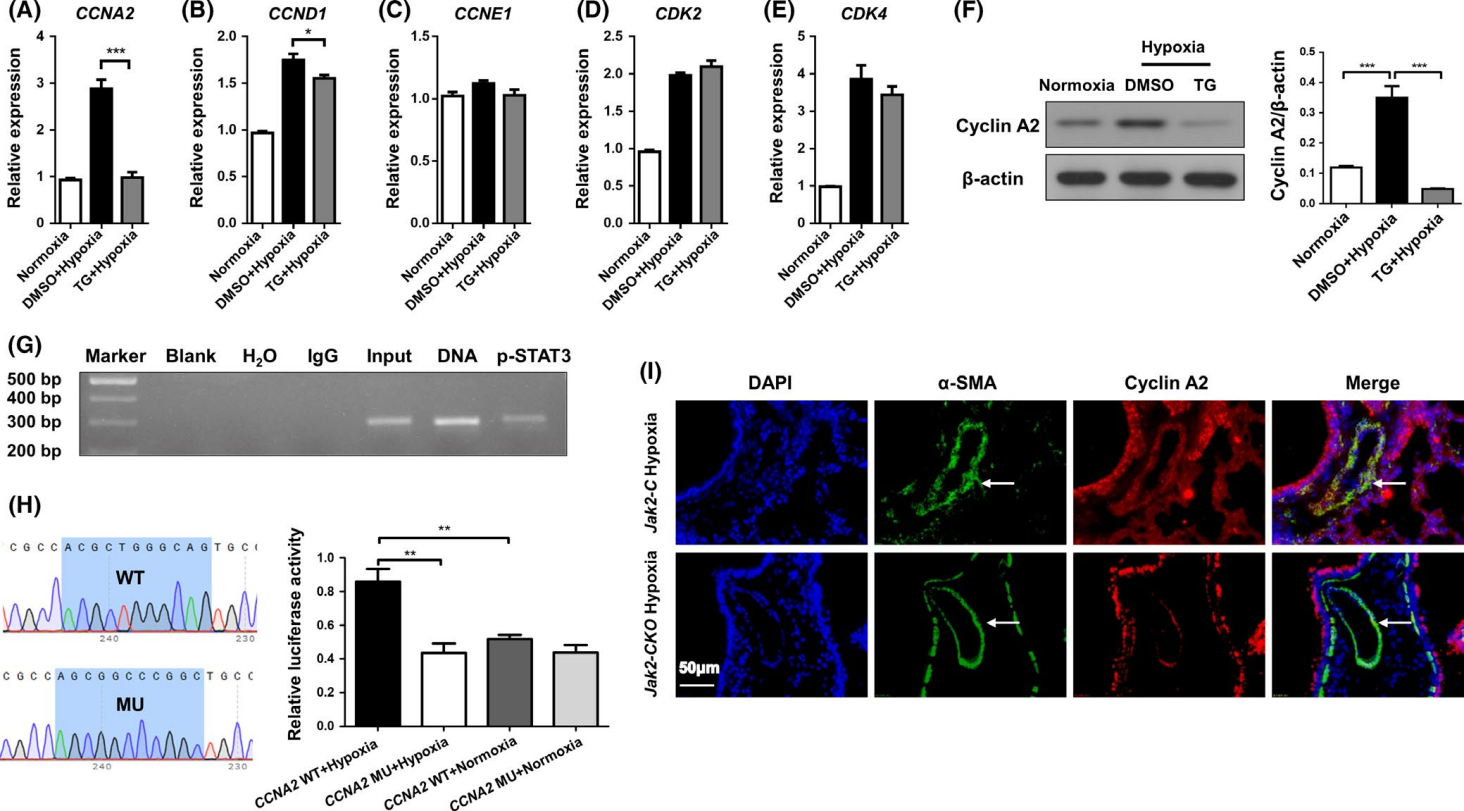
JAK2/STAT3 promoted HPASMC proliferation programming by enhancing cyclin A2 expression following hypoxic exposure. Real‐time PCR to determine cyclin A2 (A), cyclin D1 (B), cyclin E1 (C), CDK2 (D) and CDK4 (E) expression in HPASMCs. Western blot analysis of cyclin A2 expression in HPASMCs (F). ChIP‐PCR results for analysis of p‐STAT3 binding activity to the *CNNA2* promoter (G). Results for *CNNA2* promoter luciferase reporter assays in HPASMCs (H). Coimmunostaining results of α‐SMA and cyclin A2 in lung sections from *Jak2‐C* and *Jak2‐CKO* mice after hypoxic (I; n = 10 per group) exposure for 28 days. All images were taken at an original magnification of ×400. The data are represented as the mean ± SEM. *P < .05; **P < .01; ***P < .001. CDK2, cyclin‐dependent kinase 2; CDK4, cyclin‐dependent kinase 4

To dissect the mechanisms by which JAK2/STAT3 signalling promotes PASMC proliferation, we first conducted real‐time PCR analysis of cyclin A2, cyclin D1, cyclin E1, cyclin‐dependent kinase 2 (CDK2) and CDK4 expression in PASMCs, as those molecules are considered to be cell cycle checkpoint regulators for the G0/G1 and S phases.^33^ It was interestingly noted that the expression of cyclin A2, a critical regulator necessary for the initiation of cell cycle and S/G2 phase transition,^34^ was decreased by 2‐fold in TG‐treated HPASMCs as compared to that of cells treated with dimethyl sulphoxide (DMSO) under hypoxic condition (Figure 6A; 2.88 ± 0.19 vs 0.98 ± 0.11, n = 4 per group, *P* = .0001). A slight decrease of cyclin D1 was also noted (Figure 6B; 1.75 ± 0.06 vs 1.55 ± 0.035, n = 4 per group, *P* = .036), but no perceptible change was noted for the other regulators (Figure 6C‐E; cyclin E1: 1.12 ± 0.02 vs 1.03 ± 0.05, n = 4 per group, *P* = .110; CDK2: 1.98 ± 0.04 vs 2.10 ± 0.08, n = 4 per group, *P* = .248; and CDK4: 3.86 ± 0.37 vs 3.44 ± 0.23, n = 4 per group, *P* = .366). Given that cyclin D1 has been suggested to be a target for STAT3 transcription,^35^ we thus embarked on cyclin A2. Indeed, Western blot analysis confirmed a 6‐fold reduction of cyclin A2 levels in TG‐treated cells (Figure 6F; 0.35 ± 0.04 vs 0.05 ± 0.00, n = 4 per group, *P* = .0002), suggesting that cyclin A2 could be a novel target downstream of JAK2/STAT3 to regulate HPASMCs proliferation following hypoxic insults.

To address the above question, in silico analysis was conducted and identified 9 potential STAT3‐binding sites in the *CCNA2* (encoding cyclin A2) promoter (Figure S1). Chromatin immunoprecipitation (ChIP) was next conducted, and the resulting products were used to amplify the *CCNA2* promoter regions flanking the above‐indicated 9 potential p‐STAT3 binding sites. Indeed, p‐STAT3 manifested binding activity to the *CCNA2* promoter in a region 214 bp upstream of the transcriptional start site (Figure 6G). *CCNA2* promoter reporter assays were subsequently employed to confirm that STAT3 transcribes *CCNA2* expression. A mutated *CCNA2* promoter reporter (MU) was constructed in which the STAT3‐binding motif (ACGCTGGGCAG) was mutated to AGCGGCCCGGC (Figure 6H, left panel). As expected, disruption of the STAT3‐binding site resulted in a 1‐fold reduction of hypoxia‐induced luciferase reporter activity in HPASMCs (Figure 6H; right panel, 0.86 ± 0.07 vs 0.44 ± 0.06, n = 4 independent replications, *P* = .004). These findings prompted us to check cyclin A2 expression in animals induced with PAH. Consistently, *Jak2‐CKO* mice following hypoxic induction manifested remarkably lower cyclin A2 expression in PASMCs (Figure 6I). Taken together, our data support that JAK2 activates STAT3, thereby transcribing cyclin A2 expression in PASMC to enhance pulmonary blood vessel remodelling during the course of PAH development.

## 4 DISCUSSION

Although a great deal of effort has been recently devoted to dissecting the pathoaetiologies underlying PAH, the exact molecular mechanisms, however, remain poorly understood. The lack of this related information has significantly hampered the development of novel and effective therapies against this devastating disorder. There is emerging evidence that JAK/STAT signalling is activated during PAH initiation and progression. Studies in a monocrotaline (MCT)‐induced PAH model revealed that monocrotaline pyrrole (MCTP), the main metabolite of MCT, targets the pulmonary arteries to induce transforming growth factor β (TGF‐β) expression via activating JAK/STAT signalling.^36^, ^37^ TGF‐β was also noted to induce interleukin 6 (IL‐6) production through the JAK/STAT signalling cascade.^38^ Together, these data support that JAK/STAT signalling is likely implicated in the pathogenesis of PAH.

**Figure 7:**
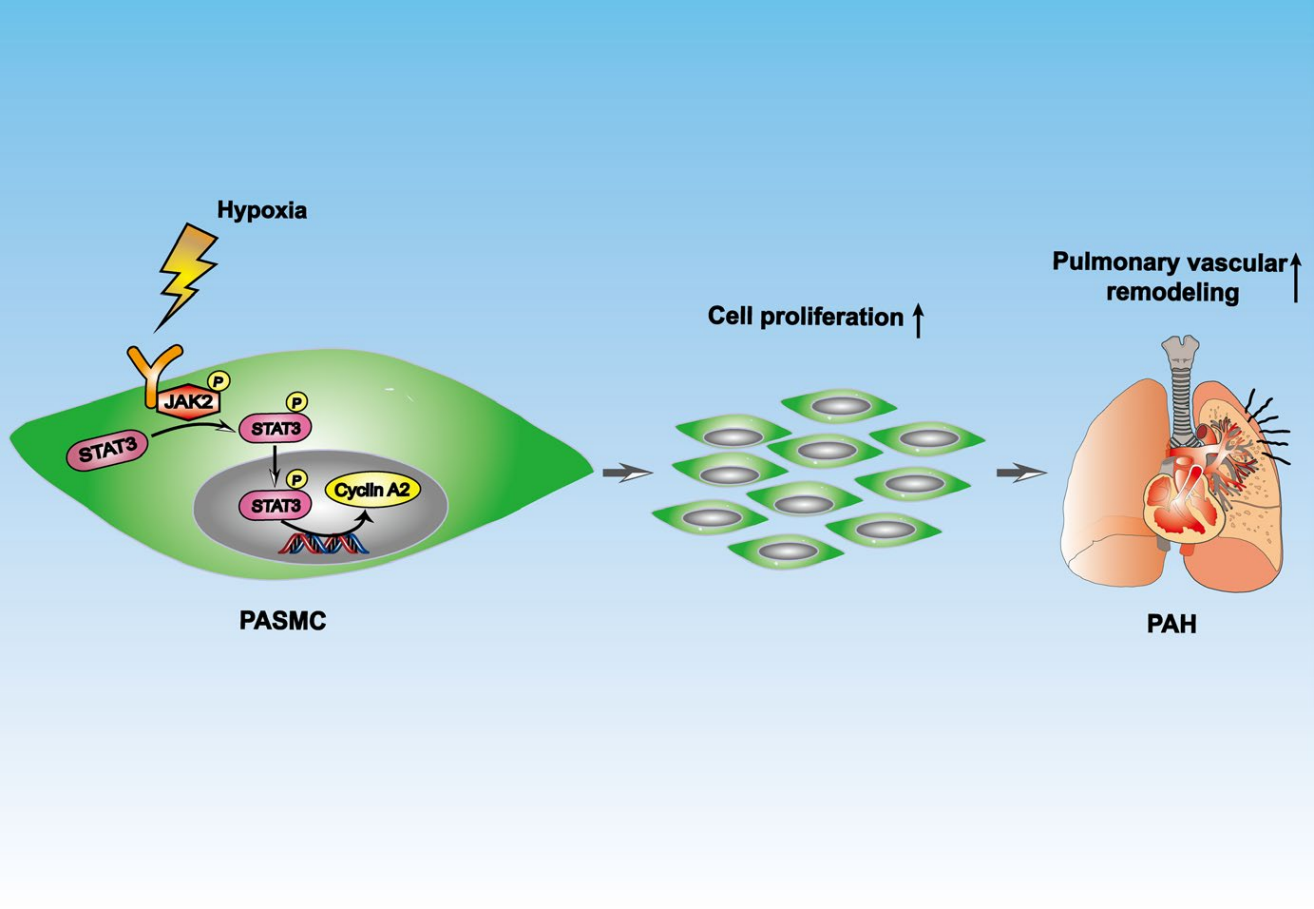
Diagram of the mechanisms underlying JAK2 regulation of PAH. Hypoxia induced PASMC proliferation through promoting the JAK2/STAT3/cyclin A2 pathway, in which STAT3 bound directly to the *CCNA2* promoter and transcriptionally activated cyclin A2 under hypoxic condition, ultimately leading to increased pulmonary arterial remodelling in PAH

In the present study, we investigated the impact of JAK2 on pulmonary blood vessel remodelling in a hypoxia‐induced PAH model. We first demonstrated chronic hypoxic insult activated JAK2/STAT3 signalling in PASMCs, and studies in HPASMCs following normobaric hypoxic exposure further confirmed the results. Therefore, depletion of *Jak2* in smooth muscle cells protected mice from chronic hypoxia‐induced pulmonary vascular remodelling and RV hypertrophy during the course of PAH development. Importantly, administration of JAK2 blocker could sufficiently reverse hypoxia‐induced HPASMC proliferation. Mechanistic studies revealed that upon hypoxia‐induced activation, JAK2 phosphorylates STAT3, which then translocates into the nucleus, where it binds to the *CCNA2* promoter to transcribe cyclin A2 expression, thereby promoting PASMC hyperplasia along with pulmonary vascular remodelling (Figure 7). These results not only provide novel insight into the pathogenesis underlying PAH, but also demonstrate evidence that blockade of JAK2 could be a viable approach to inhibit PASMC proliferation, which may have great potential for the treatment of PAH in clinical settings.

To address the above question, we challenged the mice with chronic hypoxic insult, and then examined JAK2/STAT3 signalling in the lung sections. Remarkably, the enhanced p‐JAK2 and p‐STAT3 expression was predominantly limited to PASMCs, as evidenced by the α‐SMA co‐immunostaining. Similar results were also obtained from studies in HPASMCs. Based on the above results, we hypothesized that depletion of *Jak2* in smooth muscle cells may confer protection for mice against hypoxia‐induced PAH. To prove this assumption, we generated inducible smooth muscle cell specific *Jak2* knockout mice, and *Jak2* deficiency was induced by tamoxifen as described at 8 weeks of age, followed by hypoxic insult for 28 days. Indeed, *Jak2‐CKO* mice displayed significantly reduced thickness of the medial layer of small PAs along with attenuated pulmonary vascular remodelling. Specifically, loss of *Jak2* in smooth muscle cells repressed hypoxia‐induced PASMC proliferation as manifested by the much less number of α‐SMA^+^Ki67^+^ cells.

To date, many studies have principally focused on the effect of aberrant JAK/STAT signalling on abnormal cancer cell proliferation. In head and neck squamous cell carcinoma, STAT3 phosphorylation is a consequence of increased IL‐6 production by tumour cells.^39^ Increased expression of the G‐CSF receptor is observed in high‐grade ovarian epithelial tumours, and cell culture experiments suggest that G‐CSF contributes to JAK/STAT activation in this disease.^40^ Given that hypoxia stimulates the proliferation of PASMCs, thereby promoting pulmonary blood vessel remodelling,^41^ we thus utilized TG101043 (TG), a selective JAK2 inhibitor, to elucidate the effect of JAK2 on PASMC proliferation following hypoxic insult. Excitingly, blockade of JAK2 efficiently suppressed hypoxia‐induced HPASMC proliferation, suggesting that blockade of JAK2 could be a viable approach to prevent pulmonary vascular remodelling in clinical settings.

To further address the mechanisms by which JAK2 regulates PASMC proliferation following hypoxic exposure, we first examined the effects of JAK2 inhibition on the cell cycle in HPASMCs after hypoxic challenge. Notably, compared to control cells, TG treatment significantly decreased the number of HPASMCs in G2/M phase by inducing G0/G1 and S phase arrest following hypoxic insult. This result prompted us to examine key regulators relevant to G0/G1 to S phase and S phase to G2/M phase transition after hypoxic challenge. Cyclin A2 was noted to be significantly downregulated following TG treatment. In general, cyclin A2 is not only required for G0/G1 to S phase transition, but also essential for DNA synthesis in S phase,^42^ and therefore, aberrant cyclin A2 expression would arrest the cells in G0/G1 and S phase.^43^ In fact, cyclin A2 has been noted to be involved in astrocyte proliferation in a gliosis animal model^44^ and trophoblasts in recurrent miscarriage.^45^ We thus assumed that cyclin A2 is a downstream target of JAK2/STAT3 signalling. Indeed, ChIP assays demonstrated that p‐STAT3 selectively binds to the *CCNA2* promoter at a position 214 bp upstream of the transcriptional start site, thereby transcribing cyclin A2 expression to promote PASMC proliferation following hypoxic insult. In line with these findings, *JAK2‐CKO* mice exhibited a substantial decrease in terms of cyclin A2 expression in PASMCs following chronic hypoxic exposure. Beyond cyclin A2, TG treatment also induced a slight decrease of cyclin D1 expression in HPASMCs following hypoxic exposure, while cyclin D1 has been considered to be a potential STAT3 target to mediates platelet‐derived growth factor (PDGF)‐induced proliferation in human airway smooth muscle cells.^46^, ^47^

In conclusion, we demonstrated evidence that altered of JAK2 activity in PASMCs is a critical manifestation relevant pulmonary vascular remodelling during the course of PAH development. Therefore, mice with SMC‐specific *Jak2* deficiency are protected from hypoxia‐induced pulmonary vascular remodelling and RV hypertrophy. Upon hypoxia‐induced activation, JAK2 phosphorylates STAT3, which transcribes cyclin A2 expression by binding to the *CCNA2* promoter, thereby promoting PASMC proliferation. Collectively, our data suggest that JAK2 could be a viable target to prevent blood vessel remodelling in clinical settings.

## CONFLICT OF INTEREST

The authors declare that they have no competing interests.

## AUTHORS' CONTRIBUTIONS

LZ, YW, GRW, LZR, YQW, HHY, TY, PY, FX and QZ performed experiments; SZ, JXL, BWM, HLZ, WNX and CYW designed experiments, analysed data and supported the preparation of the manuscript; and CYW led the investigation and wrote the manuscript.

## ETHICAL APPROVAL

All studies were conducted in accordance with NIH guidelines and approved by the Animal Care and Use Committee (ACUC) of Tongji Hospital. Additionally, all animals were handled with care and euthanized humanely during the study.

## Data Availability

The data that support the findings of this study are available from the corresponding author upon reasonable request.
