# Supplementary figures and images for: Blockade of JAK2 protects mice against hypoxia‐induced pulmonary arterial hypertension by repressing pulmonary arterial smooth muscle cell proliferation
Source: Cell Prolif. 2020 Jan 14;53(2):e12742. doi: 10.1111/cpr.12742 (PMC7046303; doi:10.1111/cpr.12742)

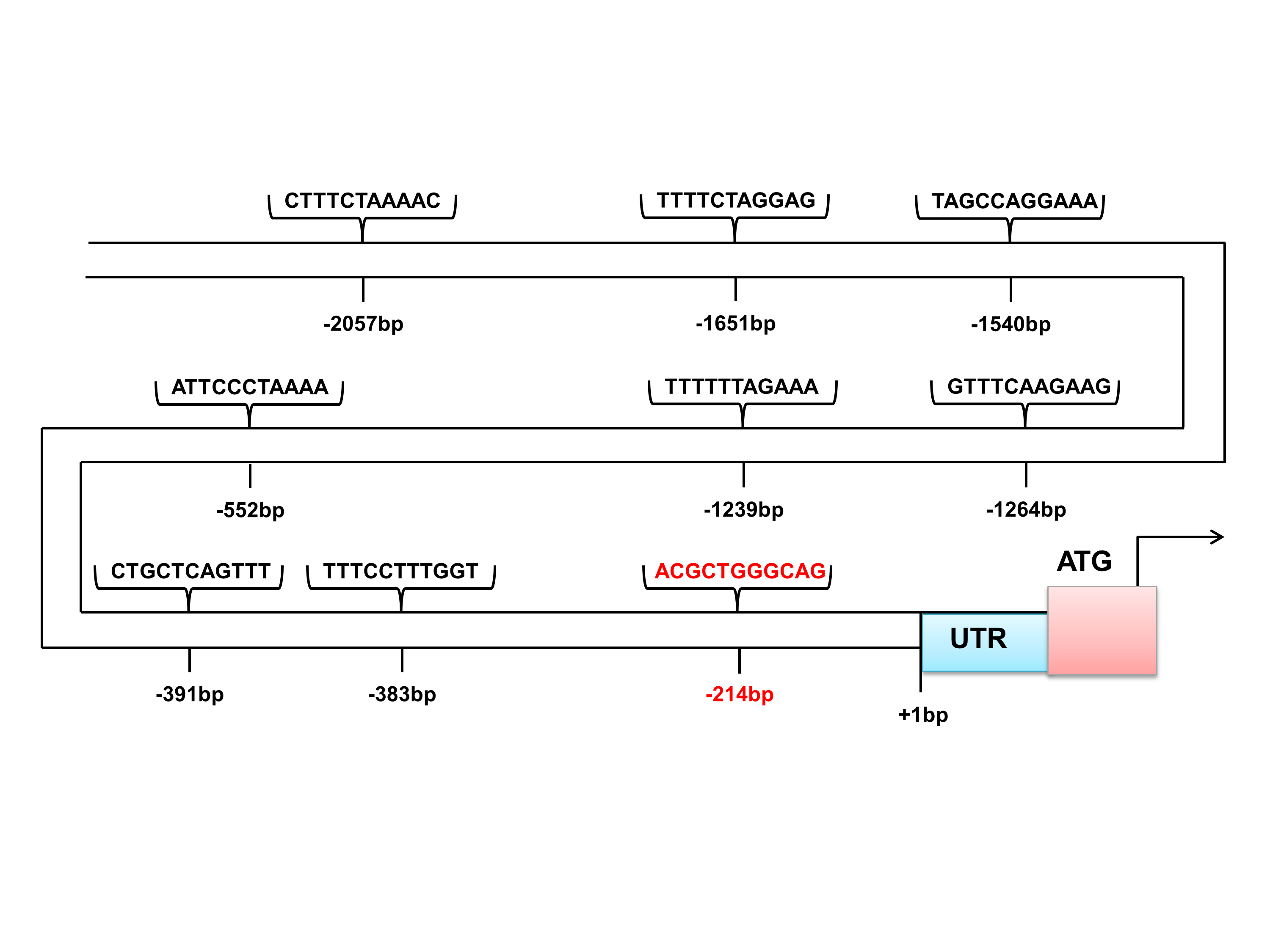

Supplement: Supplementary file 1 [file CPR-53-e12742-s001.tif]
